# Lights camera action: Randomized control trial evaluating the impact of a preoperative instructional video on patient satisfaction following minimally invasive gynecologic surgery

**DOI:** 10.1016/j.pecinn.2026.100465

**Published:** 2026-03-11

**Authors:** Sarah Simko, Nayo Animasaun, Herlinda Bergman, Angelina Lam, Michelle Porche, Janet Cruz, Mallory Stuparich, Samar Nahas

**Affiliations:** a University of California, Riverside, School of Medicine, Minimally Invasive Gynecologic Surgery, Riverside, USA; b University of California, Riverside, Department of Internal Medicine, Riverside, USA; c University of Riverside, School of Medicine, USA

**Keywords:** Minimally invasive gynecologic surgery, Video instruction, Patient education, Patient satisfaction, Pre-procedural anxiety, Patient preparedness

## Abstract

**Objectives:**

To evaluate the impact of a pre-operative instructional video for patients undergoing minimally invasive gynecologic surgery (MIGS) on patient satisfaction following surgery, pre-procedure anxiety, postoperative pain, and patient preparedness.

**Methods:**

A randomized control trial was performed at an academic subspecialty gynecologic clinic. A total of 57 patients participated in the study who underwent robotic or laparoscopic surgery with same day discharge after surgery between October 2023 and March 2025. Patients were randomly assigned to 2 groups: preoperative video in addition to standard physician counseling and standard physician counseling alone prior to surgery.

**Results:**

Patients who received supplemental video counseling had higher satisfaction scores compared to those in the control group. No differences were seen in preparedness levels, pre-procedural anxiety, medication compliance, narcotic use, postoperative pain, number of postoperative visits, or number of MyChart and telephone encounters.

**Conclusions:**

Video counseling can be utilized to improve patient satisfaction for gynecologic surgeries. Future considerations include continued development of these tools and further study on their potential benefits for improved clinical efficiency.

**Innovation:**

As technology evolves, we can continue to advance the care patients receive in the perioperative period. Audiovisual instruction can help provide patients with a visually appealing option to guide them through the steps of the recovery process and may be able to improve efficiency in the clinic visit.

## Highlights

• Administration of a preoperative counseling video prior to gynecologic surgery led to improved satisfaction and patient experience factors.
• No differences were seen in preparedness, pre-procedural anxiety, pain scores, medication compliance, or number of postoperative visits.
• Audiovisual counseling can provide standardized information to patients and can be iteratively developed based on patient feedback.

## 1 Introduction

Minimally invasive gynecologic surgery (MIGS) has become the standard of care in the treatment of many benign and malignant gynecologic conditions [1]. Minimally invasive surgical approaches have been shown to have decreased length of hospital stay, decreased opioid use, and increased patient satisfaction when compared to the open approach [2]. However, any surgical procedure can be a source of anxiety for patients which may stem from feelings of unpreparedness and lack of preprocedural education. Preoperative anxiety has been shown to increase the risk of poor wound healing, increase the length of hospital stay, and contribute to suboptimal pain control [[3], [4]]. For these reasons, it is important to evaluate techniques to help increase patient satisfaction and preparedness and decrease pre-procedural anxiety.

The effectiveness of various methods to mitigate patient anxiety before surgery and increase patient satisfaction have been studied in patients undergoing cesarean sections and bariatric surgery. These studies have shown that the addition of learning materials such as a preoperative video, may be more effective in increasing patient preparedness than relying on standard physician counseling alone [[5], [6], [7]]. Audio visual education has been found to increase short-term memory knowledge and may be a useful tool for patients shortly before having surgery when compared to verbal or written delivery of information [[8], [9]]. There are very few studies that evaluate the use of a preoperative informational video in the setting of minimally invasive gynecologic surgeries. In this study, we aimed to assess the impact of a preoperative video on patient satisfaction, patient experience factors, patient preparedness, preoperative anxiety, number of MyChart and telephone encounters postoperatively, number of postoperative appointments and postoperative pain for patients undergoing gynecologic robotic or laparoscopic surgeries.

## 2 Methods

A randomized control trial was performed between October 2023 to April 2025 of patients undergoing minimally invasive gynecologic procedures at academic-affiliated community hospitals in Riverside, California. The study was conducted in accordance with the University of California at Riverside (UCR) Institutional Review Board (approved August 9, 2023)**.** Inclusion criteria included English-speaking patients aged 18–65 years old with a benign gynecologic condition who were scheduled to undergo robotic or laparoscopic surgery at our institution with same day discharge. Exclusion criteria included patients receiving postoperative chemotherapy in the 6-week postoperative period, planned laparotomy, urogynecology procedure, and pregnancy.

Participants who met study criteria were approached in the clinic on the day of their preoperative appointment either in person or virtually. Patients were enrolled by the study personnel only. Those who agreed to participate were consented and randomized through REDCap to the invention group and were asked to watch an eight-minute preoperative counseling video in addition to standard physician counseling, versus the control group who received standard physician counseling alone. Standard physician counseling included discussion of the risks and benefits of the procedure, information on postoperative medications, and information about expectations for the postoperative course. The video included information on preoperative planning, postoperative expectations and possible symptoms, postoperative pain instructions, activity restrictions, and return precautions. Simple randomization was performed to allocate the participants into either group. Masking of care providers was performed; however, study participants and the study team were not masked. Per standard practice at our institution all patients received a written information sheet with postoperative care instructions and a phone call from a physician on postoperative day 1. All patients were asked to complete a preliminary survey to assess demographics, prior medical and surgical history, preoperative substance use, and preoperative baseline pain. Preliminary questions were adapted from the CAHPS® Surgical Care Survey. Those in the intervention group were provided with a link to view the preoperative video through REDCap and were asked if they watched the video in full at the end of the video. Patients were able to watch the video as many times as they desired.

The primary outcome of this study was patient satisfaction following the procedure in the intervention versus the control group. Secondary outcomes included the level of patient preparedness, pre-procedural anxiety, patient experience, compliance with pain medication regimen utilized following the procedure, postoperative pain levels, number of postoperative visits, number of telephone and MyChart encounters and medication refill requests between groups. This study also aimed to assess if various factors including history of prior surgeries, postoperative complications, preoperative pain scores, and preoperative substance use, among others had an impact on these outcomes.

All patients received a link to complete a postoperative questionnaire at or after their two-week postoperative follow-up appointment. This questionnaire used standardized measures to assess for various outcomes. Postoperative patient satisfaction was assessed using the Surgical Satisfaction Questionnaire (SSQ-8) and a numeric satisfaction score (NSS) with patients ranking satisfaction on a scale of 1 to 100 (with 100 signifying max satisfaction) [Appendix A]. SSQ-8 scores were normalized from 0 to 100, where 100 represents the highest level of satisfaction. Question 4 of the SSQ-8 survey asks patients about their satisfaction levels with the time it took them to return to work. In cases in which a patient selected Not Applicable (N/A) as an answer for this question, the total score was divided by the total number of questions answered. Analysis was also performed by substituting in neutral responses for those who selected N/A for a response, as well as by removing those participants from the analysis. Cut offs were based off the mean scores.

Preoperative anxiety was assessed using the Amsterdam Preoperative Anxiety and Information Scale (APAIS) and a numeric anxiety score from 1 to 100 [Appendix A]. Based on prior cut-offs, a score of 11 or greater was utilized for the anxiety subset, which summed items 1, 2, 4, and 5. A score of 8 or greater was utilized for the information subset, which summed items 3 and 6. Patient experience was measured using Likert scale questions from the CAHPS® Surgical Care Survey [Appendix A]. Top box scoring was used for the analysis. Patient preparedness was measured using a numeric score between 1 and 100. Patients were also asked what would have many them feel more prepared preoperatively. Pre- and post-operative pain were assessed using a baseline pain score from 1 to 5 and an assessment of the patient's worst pain level from 1 to 5 (with 5 signifying the highest pain level). Patients were counseled to take nonsteroidal anti-inflammatory drugs (NSAIDs) and acetaminophen in a scheduled fashion following the surgery and an opioid medication as needed. Compliance with these instructions was assessed in the postoperative survey as well as the number of opioid refill requests and the opioid requirements in each group. In our practice, patients are generally prescribed approximately 10–15 pills of a narcotic medication. Those who required greater than 10 pills were compared against those who required 10 or fewer pills.

Following completion of the surveys, chart review was performed to assess for age, body mass index (BMI), preoperative diagnosis, procedure performed, mini-laparotomy, surgical approach, intraoperative and postoperative complications, admissions and readmissions, emergency department visits, and the number of postoperative appointments. The number of MyChart and telephone encounters within the first two weeks postoperatively for a medical concern was also assessed as well as the reason for the encounter. These were categorized into those who had 1 or more telephone or MyChart encounters versus those who had no encounters. Patients with one postoperative visit, which is standard in our practice for routine care were compared against those who had greater than one postoperative appointment. BMI was categorized into two groups: BMI < 30 and BMI ≥ 30. Age was categorized into age < 40 and age ≥ 40. Complications were included if they were Clavien-Dindo Classification Grade 1 or higher with the thought that any deviation from the normal postoperative course could affect patient satisfaction, pain scores and the number of postoperative visits. The Clavien-Dindo Classification Scale is a tool used to categorize surgical complications based on the severity of treatment required. Readmissions and emergency department visits within 2 weeks of the procedure were included.

Race and ethnicity were self-identified by the partipants; however, these were categorized into Non-Hispanic Black, Non-Hispanic White, Non-Hispanic Asian, Hispanic, and Multiple Races for analysis. Sexual Orientation was categorized into Heterosexual, LGBTQAI+ and Missing. Educational status was categorized into less than a high school diploma or high school graduate or greater. Overall health and mental health scores were assessed using a Likert scale from 1 to 5 and were categorized into scores greater than or equal to 3 or less than 3. Patients were asked about the presence of concurrent pain syndromes and those with greater than 1 comorbid pain condition were compared against those without [see Appendix 1].

The sample size calculation was based on the primary outcome of SSQ-8 patient satisfaction score. Assuming a two-sided alpha of 0.05, power of 80%, and an effect size of 0.5, a total sample size of 52 participants was required. No adjustment was made for attrition. Statistical analyses were performed using chi-squared tests for categorical variables. An intention to treat analysis was performed. Missing values were removed from the analysis. All subgroup and sensitivity analyses were exploratory and not pre-specified unless otherwise stated. A thematic analysis was performed to assess the primary areas where patients felt they could have been better prepared. Participants' responses were coded and analyzed. These responses were read and codes were generated based on meaning making sections of the quotes. These codes were then reviewed and five themes were identified that described the patients' perspectives.

## 3 Results

**Fig. 1:**
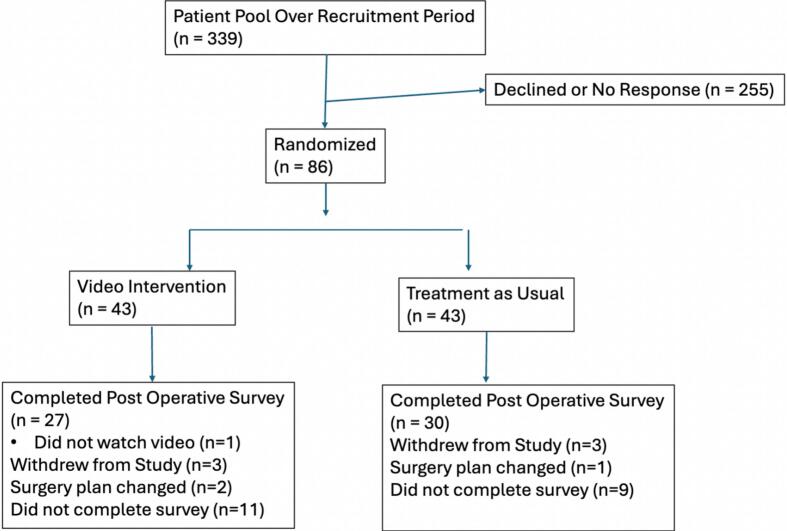
CONSORT (Consolidated Standards of Reporting Trials) Flow Diagram.

Eighty-six patients agreed to participate in the study with 57 being consented and completing the postoperative survey [Fig. 1]. There were 30 patients in the verbal instruction only group and 27 patients in the video plus verbal instruction group. 29 participants were withdrawn from the study, including 2 patients whose surgical plan changed, 1 patient who had their surgery outside of our institution, 6 patients who withdrew their consent, and 20 patients who did not complete the questionnaires. One patient did not watch the preoperative video. An intention to treat analysis was performed.

There were no demographic differences between the two groups [Table 1]. The majority of patients identified as Hispanic, heterosexual, with an average age of 40. Most patients had completed high school. The majority of patients presented for a primary complaint of pelvic pain and underwent a minimally invasive hysterectomy. Complications including 2 urinary tract infections, 4 surgical infections, 1 transfusion, 1 bladder injury, 2 cases of acute urinary retention, 3 minor allergic reactions. Three participants presented to the emergency room for constipation, deep venous thrombosis (DVT) rule out and an allergic reaction. Most patients reported good overall and mental health at baseline. Average baseline pain scores were greater than 3, and most patient reported at least one chronic pain condition.

**Table 1:** Demographics of study participants.

	Verbal	Verbal + Video	*P*-value
**Age**			
<40	13 (43%)	14 (52%)	0.52
≥40	17 (57%)	13 (48%)	
**BMI**			
<30	11 (38%)	13 (48%)	0.44
≥30	18 (62%)	14 (52%)	
**Race/Ethnicity**			
Non-Hispanic Black	5 (16%)	4 (15%)	0.64
Non-Hispanic White	8 (26%)	7 (27%)	
Non-Hispanic Asian	2 (6%)	0 (0%)	
Hispanic	13 (43%)	15 (56%)	
Multiple Races	2 (6%)	1 (4%)	

**Sex at Birth**			
Female	30 (100%)	27 (100%)	N/A
Gender			
Woman	30 (100%)	27 (100%)	N/A
**Sexual Orientation**			
Heterosexual	26 (87%)	23 (85%)	0.089
LGBTQAI+	0 (0%)	3 (11%)	
Missing	4 (13%)	1 (4%)	

**Educational Status**			
Less than secondary	0 (0%)	2 (7%)	0.13
High school graduate or greater	30 (100%)	25 (93%)	

**Primary Indication for Surgery**			
Abnormal Uterine Bleeding	7 (23%)	3 (11%)	0.24
Pelvic Pain	20 (63%)	17 (67%)	
Adnexal Mass	3 (10%)	3 (11%)	
Fibroids	0 (0%)	3 (11%)	
Sterilization	1 (3%)	0 (0%)	

**Primary Procedure**			
Hysterectomy	16 (53%)	13 (48%)	0.72
Excision of Endometriosis	11 (37%)	11 (41%)	
Adnexal Surgery	3 (10%)	2 (7%)	
Myomectomy	0 (0%)	1 (4%)	

Mini-Laparotomy	3 (10%)	5 (19%)	0.36
Robotic	17 (57%)	18 (67%)	0.44
Complication	4 (13%)	8 (30%)	0.13
Admit	0 (0%)	0 (0%)	N/A
Readmit	0 (0%)	1 (4%)	0.29
ED Visit	3 (10%)	0 (0%)	0.09

Overall Health ≥ 3	23 (79%)	24 (89%)	0.32
Mental Health ≥ 3	22 (76%)	22 (81%)	0.61
Preoperative Narcotics Use	8 (29%)	4 (15%)	0.24
Preoperative Baseline Pain ≥ 3	17 (63%)	17 (76%)	0.74
Preoperative Worst Pain ≥ 3	22 (78%)	21 (78%)	0.87
Comorbid Chronic Pain Conditions > 1	16 (53%)	13 (48%)	0.70

Patients in the intervention group had higher SSQ-8 scores compared to those in the control group (*p* = 0.035) [Table 2]. While all participants completed the SSQ-8 questionnaires, five (19%) participants in the video group and eight (27%) participants in the verbal group selected N/A when asked about their satisfaction returning to work (*p* = 0.46). As mentioned previously, primary analysis was performed by dividing the total score by the total number of questions answered. Sensitivity analysis by substituting neutral scores for those who selected N/A as a response showed a similar result with higher SSQ-8 scores in the video group compared to the verbal group (*p* = 0.019). When these participants were removed from the analysis, a significant difference was no longer noted between the two groups (*p* = 0.26). NSS were similar between the two groups. Patient experience measures showed higher ratings of experience in the video group on certain measures. Patients in the video group were 30% more likely to report that they had received easy to understand instructions about getting ready for surgery (*p* = 0.0073) and 33% more likely to report being counseled on signs or symptoms that would need immediate medical attention during the recovery period (*p* = 0.031).

**Table 2:** Postoperative outcomes in the verbal versus the verbal and video group.

	Verbal	Verbal + Video	P-Value
SSQ-8 score ≥ 80	18 (60%)	23 (85%)	0.035
NSS ≥ 80	28 (100%)	25 (93%)	0.14
CAHPS1 = 3	29 (93%)	24 (93%)	0.91
CAHPS2 = 3	23 (77%)	27 (100%)	0.0073
CAHPS3 = 3	23 (77%)	23 (85%)	0.42
CAHPS4 = 3	21 (70%)	25 (93%)	0.031
CAHPS5 = 3	22 (73%)	22 (81%)	0.46
CAHPS6 = 3	27 (81%)	23 (90%)	0.58

Preparedness Numerical Scale ≥ 95	18 (62%)	22 (85%)	0.061

Anxiety Subset ≥ 11	9 (30%)	10 (37%)	0.57
Information Subset ≥ 8	10 (33%)	10 (37%)	0.77
Anxiety Numerical Scale ≥ 80	10 (36%)	9 (33%)	0.85
**Medication Compliance**			
Tylenol	18 (58%)	16 (62%)	0.95
Ibuprofen	18 (60%)	17 (63%)	0.82
Opioid	26 (87%)	21 (78%)	0.38
**Opioid Requirements**			
Required>10 pills	5 (17%)	3 (11%)	0.55
Opioid Refill	1 (3%)	1 (4%)	0.94
Postoperative Baseline Pain	20 (67%)	18 (67%)	1.00
Postoperative Worst Pain	28 (93%)	22 (81%)	0.17
Number of Postoperative Visits > 1	11 (37%)	7 (26%)	0.38
Number of Phone and MyChart Encounters ≥ 1	15 (50%)	13 (48%)	0.89

Abbreviations: SSQ-8- Surgical Satisfaction Questionnaire 8, NSS- Numerical Satisfaction Score, CAHPS- Consumer Assessment of Healthcare Providers and Systems.

When an intention to treat analysis was performed, there was no difference in preparedness scores (*p* = 0.061). However, when a subset analysis was performed looking at participants who watched the video (*n* = 26) versus those who did not (*n* = 31), the video group had higher numerical preparedness scores than the control group (*p* = 0.02). No other statistically significant differences were observed in this subset analysis. There were no differences in preprocedural anxiety scores, medication compliance, narcotic use, postoperative pain, number of postoperative visits, or number of MyChart and telephone encounters.

Of the 70% of patients who replied when asked if there were any areas they felt that they could have been better prepared for surgery, 63% responded that there were no areas to improve. Among the 37% who provided feedback, the following themes emerged: More specific information about the recovery process on activity restrictions, management of pain and constipation, and voiding expectations, Preoperative anxiety management, Clinic Communication, and Setting expectations for possible complications. On review of the most frequently assess questions during myChart and telephone encounters, questions arose regarding incisional issues, pathology and surgical findings, postoperative restrictions, vaginal bleeding, postoperative pain, menopausal symptoms, constipation, urinary issues, medication issues, non-gynecology medical concerns, nausea, and allergic reactions.

## 4 Discussion and Conclusion

### 4.1 Discussion

This study demonstrated that patients who watched a preoperative counseling video in addition to standard physician counseling reported higher satisfaction scores. Patient experience factors were improved in the video group with patients being more likely to state that they received easy to understand instructions and information regarding return precautions. No differences were observed in preparedness, pre-procedural anxiety, patient phone calls to the clinic, or postoperative pain.

While few studies have assessed the impact of a preoperative counseling video on patient satisfaction for patients undergoing minimally invasive gynecologic procedures, studies in other fields have demonstrated improved satisfaction scores for patients receiving video instruction. One study that assessed the impact of preoperative video information on patient satisfaction and pre-procedural anxiety for patients undergoing open abdominal surgeries demonstrated improved satisfaction scores and decreased pre-procedural anxiety scores [, 2019]. A study assessing the impact of multimedia counseling, which included video education, compared to standard care for patient undergoing endometrial cancer staging surgery showed increased patient satisfaction in the video group [10]. Additionally, a quasi-experimental study of 32 patients showed increased patient understanding and patient satisfaction following video assisted-informed consent [11]. This discussed that personalized verbal explanations were needed to address patient's specific questions following the video. Unlike these study, two studies assessing the impact of patients receiving audiovisual materials to improve informed consent for hysterectomy showed there was no difference in patient satisfaction [[12], [13]]. The information in these videos; however, focused on the indications and routes for hysterectomy and the risks, benefits, and alternatives to hysterectomy as opposed to postoperative and recovery expectations.

Similar to this study, no differences were seen in pre-procedural anxiety following hysterectomy in those who did or did not receive video instruction [12]. Several studies in various fields have shown that patients who watched a preoperative video in addition to physician interview had a reduced level of uncertainty regarding their procedure compared to those who received information from their physician via interview alone or interview plus written material [[14], [15], [16], [17]]. The APAIS, State-Trait Anxiety Inventory (STAI), and Anxiety Specific to Surgery Questionnaire (ASSQ) questionnaires were administered preoperatively in these studies versus postoperatively in our study, which could have an impact on patient's responses.

While there was no difference in patient preparedness in this study in the intention to treat group, higher patient preparedness scores were noted in a subset analysis comparing those who watched the video versus those who did not. Given the smaller sample size of this study and the high levels of patient preparedness in both groups, this study may not have been adequately powered to assess for small differences between the groups. Patients in the control group had overall high preparedness scores, which may have made it more difficult to tease out significant differences between the two groups regarding preparedness level and other measures. This study also utilized a numerical preparedness scale, which may not have been as sensitive an assessment for preparedness. Similarly, a study of patients undergoing pelvic reconstructive surgery who underwent video counseling versus usual care showed no difference in patient preparedness [18]. This study found that the perception of increased time the health care team spent with the patient was associated with greater preparedness, although not actual time spent.

In this study we assessed in what areas patients could have felt better prepared. Areas for future improvement in preoperative counseling include continuing to adapt the video to incorporate some of this patient feedback; for example, incorporating more specific information on constipation management and voiding symptoms as well as anticipatory counseling on possible variations in the postoperative course.

Patient experience factors were found to increase in some but not all areas in this study. Patients in the video group were more likely to report that they received easy to understand information prior to the surgery and information on signs or symptoms that would need immediate medical attention during the recovery period. These findings suggest that audiovisual counseling can help provide more standardized information to patients, allowing them to feel more informed prior to surgery.

### 4.2 Limitations

Despite its strengths, this study has some limitations. Questionnaires are known to be subjective in nature and susceptible to self-report bias, although standardized surveys were incorporated to reduce the risk of bias. In addition, the sample size is small and may not being generalizable and representative of the broader population. Additionally, the study may not be powered to assess for smaller differences in the secondary outcomes. This study was also designed to assess outcomes within a short period of time (2 weeks) and so the long-term effects of our intervention may not be captured adequately. While the two study groups were similar in the compared factors, there may be alternative confounders that were not controlled for. While there was no statistically significant difference in complication rates, there was a trend towards higher complications in the video group, which could have had an impact on the patient's responses postoperatively. Additionally, this study assessed patients who were undergoing various procedures and with various preoperative diagnoses which may have introduced bias or influenced preoperative anxiety levels; although there were no significant differences in the types of procedures or preoperative diagnosis between the two groups.

### 4.3 Innovation

This study is one of the first to assess the impact of a preoperative counseling video on patient satisfaction, preparedness for surgery, and preprocedural anxiety for patients undergoing minimally invasive gynecologic surgeries. While we cannot control for all confounders, we attempted to minimize the risk of bias by comparing various confounders between the two groups, including complications, ED visits, baseline pain, among others.

The perioperative period can be a stressful time for patients and it can be difficult to retain the laundry list of risks and benefits and to dos that are discussed during the preoperative period. Video instruction can be a safe and effective method to augment patients' experience and provide them with easily understandable instructions. While this study used video counseling to supplement usual care, future research could examine whether patient satisfaction remains high when parts of the counseling are shifted entirely to video, with the aim of standardizing patient education and improving clinic efficiency. Providing patients with counseling videos prior to the preoperative visit may allow them to enter the conversation more informed and prepare questions while limiting repetitive counseling by the clinician.

### 4.4 Conclusion

This study demonstrates that a preoperative counseling video can improve patient satisfaction among patients undergoing minimally invasive gynecologic surgeries. Iterative development to refine the video may further improve patient experiences. Future studies to evaluate the use of instructional videos to decrease provider counseling time and other methods to improve pre-procedure anxiety can help augment patient experiences further down the line and improve efficiency in the clinic.

## CRediT authorship contribution statement

**Sarah Simko:** Writing – original draft, Project administration, Methodology, Investigation, Formal analysis. **Nayo Animasaun:** Writing – original draft. **Herlinda Bergman:** Project administration. **Angelina Lam:** Project administration. **Michelle Porche:** Writing – review & editing, Supervision. **Janet Cruz:** Writing – review & editing, Supervision, Resources, Conceptualization. **Mallory Stuparich:** Writing – review & editing, Supervision. **Samar Nahas:** Writing – review & editing, Supervision.

## Funding

No funding sources were utilized for this study.

## Declaration of competing interest

The authors declare that they have no known competing financial interests or personal relationships that could have appeared to influence the work reported in this paper.

## Appendix A Supplementary data

Supplementary material Image 1

## References

[1] Stewart K.I., Fader A.N. (2017). New developments in minimally invasive Gynecologic oncology surgery. Clin Obstet Gynecol.

[2] Aubrey C., Nelson G. (2023). Enhanced recovery after surgery (ERAS) for minimally invasive Gynecologic oncology surgery: a review. Curr Oncol.

[3] Stamenkovic D.M., Rancic N.K., Latas M.B., Neskovic V., Rondovic G.M., Wu J.D. (2018). Preoperative anxiety and implications on postoperative recovery: what can we do to change our history. Minerva Anestesiol.

[4] Hah J.M., Cramer E., Hilmoe H., Schmidt P., McCue R., Trafton J. (2022). Factors associated with acute pain estimation, postoperative pain resolution, opioid cessation, and recovery: secondary analysis of a randomized clinical trial. J Amer Med Assoc Netw Open 2019; 2:e190168. Erratum in: JAMA Netw Open.

[5] Maghalian M., Mohammad-Alizadeh-Charandabi S., Ranjbar M., Alamdary F.A., Mirghafourvand M. (2024). Informational video on preoperative anxiety and postoperative satisfaction prior to elective cesarean delivery: a systematic review and meta-analysis. BMC Psychol.

[6] Che Y.J., Gao Y.L., Jing J., Kuang Y., Zhang M. (2020). Effects of an informational video about Anesthesia on pre- and post-elective Cesarean section anxiety and recovery: a randomized controlled trial. Med Sci Monit.

[7] Helms L.J. (2020). Video education to improve preoperative anxiety in the bariatric surgical patient: a quality improvement project. J Perianesth Nurs.

[8] Farrell E.H., Whistance R.N., Phillips K., Morgan B., Savage K., Lewis V. (2014). Systematic review and meta-analysis of audio-visual information aids for informed consent for invasive healthcare procedures in clinical practice. Patient Educ Couns.

[9] Waller A., Forshaw K., Bryant J., Carey M., Boyes A., Sanson-Fisher R. (2015). Preparatory education for cancer patients undergoing surgery: a systematic review of volume and quality of research output over time. Patient Educ Couns.

[10] Tucker, Katherine et al., A prospective randomized trial of standard versus multimedia-supplemented counseling in patients undergoing endometrial cancer staging surgery. Gynecol Oncol, Volume 166, Issue 3, 397–402. 10.1016/j.ygyno.2022.07.013 35863993

[11] Suzui R., Taki M., Yamanoi K., Kitamura S., Sunada M., Okunomiya A. (2025). Usefulness of video explanation for informed consent in gynecologic surgery. Int J Gynecol Obstet.

[12] Wong A., Ellett L., Hodge T., McNamara H., Readman E., Potenza S. (2024). Seeing is believing: empowering patients through video education. A RCT evaluating a multimedia video for patients undergoing TLH. J Minim Invasive Gynecol.

[13] Pallett Alicia C., Nguyen Bao T., Klein Natalie M., Phippen Neil, Miller Caela R., Barnett Jason C. (2018). A randomized controlled trial to determine whether a video presentation improves informed consent for hysterectomy. Am J Obstet Gynecol.

[14] Ahmed K.J., Pilling J.D., Ahmed K., Buchan J. (2019). Effect of a patient-information video on the preoperative anxiety levels of cataract surgery patients. J Cataract Refract Surg.

[15] Soydaş Yeşilyurt D., Yildiz Findik Ü. (2019). Effect of preoperative video information on anxiety and satisfaction in patients undergoing abdominal surgery. Comput Inf Nurs.

[16] Bozkurt M., Erkoc M., Can O., Danıs E., Canat H.L. (2023). The effect of an information video on preoperative anxiety level before percutaneous nephrolithotomy procedure: a prospective, randomized trial. Can Urol Assoc J.

[17] Karalar M., Demirbas A., Gercek O., Topal K., Keles I. (2023). Impact of preoperative video-based education on anxiety levels in patients with renal stones scheduled for flexible Ureteroscopic lithotripsy: a comparative study using APAIS and STAI. Med Sci Monit.

[18] Greene K.A., Wyman A.M., Scott L.A., Hart S., Hoyte L., Bassaly R. (2017). Evaluation of patient preparedness for surgery: a randomized controlled trial. Am J Obstet Gynecol.

