# Supplementary material for: Lights camera action: Randomized control trial evaluating the impact of a preoperative instructional video on patient satisfaction following minimally invasive gynecologic surgery
Source: PEC Innov. 2026 Mar 11;8:100465. doi: 10.1016/j.pecinn.2026.100465 (PMC13014906; doi:10.1016/j.pecinn.2026.100465)
Supplement: Supplementary file 1 — Supplementary material [file mmc1.docx]

**Appendix A: Preoperative and Postoperative Surveys**

**Preoperative Survey:**

**Age:**

**Ethnicity**

- Hispanic or Latino
- Not Hispanic or Latino
- Unknown / Not Reported

**Race**

- American Indian/Alaska Native
- Asian
- Native Hawaiian or Other Pacific Islander
- Black or African American
- White
- More Than One Race
- Unknown / Not Reported

**Sex at Birth**

- Female
- Male
- Other

**Gender**

- Female
- Male
- Nonbinary
- Other
- Prefer not to answer

**Sexual Orientation**

- Lesbian
- Gay
- Bisexual
- Straight
- Other
- Prefer not to answer

**Highest grade or level of school that you have completed?**

- 8th grade or less
- Some high school, but did not graduate
- High school graduate or GED
- Some college or 2-year degree
- 4-year college graduate
- More than 4-year college degree

**In general, how would you rate your overall health?**

- Excellent
- Very good
- Good
- Fair
- Poor

**In general, how would you rate your overall mental or emotional health?**

- Excellent
- Very good
- Good
- Fair
- Poor

**Not counting this surgery, about how many other surgeries have you had?**

- None
- 1 surgery
- 2 surgeries
- 3 to 5 surgeries
- 6 to 9 surgeries
- 10 or more

**Smoking status (Tobacco)**

- Current smoker
- Past smoker
- Never smoker

**Have you used narcotics within the past 12 months?**

- Yes
- No

**How many pills of a narcotic have you used in the past week?**

- None
- 1-5
- 6-10
- 11-15
- 16-20
- 21+

**How would you rate your baseline pain level in the last 2 weeks on a scale of 1-5 (1 being no pain and 5 being the worst)?**

- 1
- 2
- 3
- 4
- 5

**How would you rate your most severe pain level in the last 2 weeks on a scale of 1-5 (1 being no pain and 5 being the worst)?**

- 1
- 2
- 3
- 4
- 5

**Do you have a history of the following conditions? (check all that apply)**

- Interstitial cystitis/Painful Bladder Syndrome
- Fibromyalgia
- Irritable Bowel Syndrome
- Pelvic floor Dysfunction
- Fibroids
- Adenomyosis
- Endometriosis
- Abdominal myofascial pain syndrome
- Physical Abuse
- Pelvic adhesive disease
- Pelvic inflammatory disease

**Postoperative Surveys**

**SSQ8:**

**How satisfied are you with how your pain was controlled in the hospital after surgery?**

- Very Satisfied
- Satisfied
- Neutral
- Unsatisfied
- Very Unsatisfied

**How satisfied are you with how your pain was controlled at home after surgery?**

- Very Satisfied
- Satisfied
- Neutral
- Unsatisfied
- Very Unsatisfied

**How satisfied are you with the amount of time it took for you to return to your daily activities, for example housework or social activities outside the house?**

- Very Satisfied
- Satisfied
- Neutral
- Unsatisfied
- Very Unsatisfied
- N/A

**How satisfied are you with the time it took you to return to work?**

- Very Satisfied
- Satisfied
- Neutral
- Unsatisfied
- Very Unsatisfied
- N/A

**How satisfied are you with the amount of time it took you to return to your normal exercise routine?**

- Very Satisfied
- Satisfied
- Neutral
- Unsatisfied
- Very Unsatisfied

**How satisfied are you with the results of your surgery?**

- Very Satisfied
- Satisfied
- Neutral
- Unsatisfied
- Very Unsatisfied

**Looking back, if you had to "do it all over again" would you have the surgery again?**

- Yes
- Maybe (Probably Yes)
- Unsure
- Don’t think so
- Never

**Would you recommend this surgery to someone else?**

- Yes
- Maybe (Probably Yes)
- Unsure
- Don’t think so
- Never

**NSS: What is your satisfaction with your surgical experience on a scale of 1–100? (100 being the most satisfied)**

**Information To Help You Prepare For Surgery**

**CAHPS1: Before your surgery, did anyone in this surgeon's office give you all the information you needed about your surgery?**

- Yes, Definitely
- Yes, Somewhat
- No

**CAHPS2: Before your surgery, did anyone in this surgeon's office give you easy to understand instructions about getting ready for your surgery?**

- Yes, Definitely
- Yes, Somewhat
- No

**Information To Help You Recover From Surgery**

**CAHPS3: Did anyone in this surgeon's office explain what to expect during your recovery period?**

- Yes, Definitely
- Yes, Somewhat
- No

**CAHPS4: Did anyone in this surgeon's office warn you about any signs or symptoms that would need immediate medical attention during your recovery period?**

- Yes, Definitely
- Yes, Somewhat
- No

**CAHPS5: Did anyone in this surgeon's office give you easy to understand instructions about what to do during your recovery period?**

- Yes, Definitely
- Yes, Somewhat
- No

**CAHPS6: Did this surgeon make sure you were physically comfortable or had enough pain relief after you left the hospital or surgical facility where you had your surgery?**

- Yes, Definitely
- Yes, Somewhat
- No

**How prepared did you feel for this surgery on a scale from 1-100 (100 being the most prepared)?**

**APAIS:**

**How worried were you about the anesthesia preoperatively?**

- Not at all worried
- Slightly worried
- Moderately worried
- Very worried
- Extremely worried

**The anesthesia was on my mind continuously, preoperatively.**

- Not at all true
- Slightly true
- Moderately true
- Very true
- Extremely true

**I wanted to know as much information as possible about the anesthesia, preoperatively.**

- Not at all true
- Slightly true
- Moderately true
- Very true
- Extremely true

**How worried were you about the surgery preoperatively?**

- Not at all worried
- Slightly worried
- Moderately worried
- Very worried
- Extremely worried

**The surgery was on my mind continuously, preoperatively.**

- Not at all true
- Slightly true
- Moderately true
- Very true
- Extremely true

**I wanted to know as much information as possible about the surgery, preoperatively.**

- Not at all true
- Slightly true
- Moderately true
- Very true
- Extremely true

**On a scale from 1 to 100, how would you rank your anxiety level before the surgery (100 being the highest anxiety)?**

**Pain Control and Uptake of Instructions:**

**How often did you use the Tylenol you were prescribed in the first week after surgery?**

- Scheduled around the clock, every 6-8 hours
- As needed for pain
- 1-2 times
- Not at all
- Unsure

**How often did you use the Ibuprofen you were prescribed in the first week after surgery?**

- Scheduled around the clock, every 6-8 hours
- As needed for pain
- 1-2 times
- Not at all
- Unsure

**How often did you use the opioid (Oxycodone, Norco) you were prescribed in the first week after surgery?**

- Scheduled around the clock, every 6-8 hours
- As needed for pain
- 1-2 times
- Not at all
- Unsure

**How many pills of the opioid (Oxycodone, Norco) did you require after the surgery at home?**

- None
- 1-5
- 6-10
- 11-15
- 16-20
- 21+

**How would you rate your baseline pain after the surgery in the first week on a scale of 1-5 (1 being no pain and 5 being the worst)?**

- 1
- 2
- 3
- 4
- 5

**How would you rate your worst pain after the surgery in the first week on a scale of 1-5 (1 being no pain and 5 being the worst)?**

- 1
- 2
- 3
- 4
- 5
